# Neuropeptide Y Boosts Intestinal Mucosal Immunity of Tilapia Infected with *Streptococcus agalactiae* by Reducing Inflammation and Oxidative Stress

**DOI:** 10.3390/ani15182730

**Published:** 2025-09-18

**Authors:** Yang Yu, Ziyan Liu, Mengyuan Zhou, Zexia Chen, Ran Cai, Chaowei Song, Meiqing Li, Tiansheng Zhu, Caiyun Sun, Wensheng Li

**Affiliations:** State Key Laboratory of Biocontrol, Guangdong Province Key Laboratory for Aquatic Economic Animals, Guangdong Provincial Engineering Technology Research Center for Healthy Breeding of Important Economic Fish, School of Life Sciences, Sun Yat-Sen University, Guangzhou 510275, China

**Keywords:** NPY family, *Streptococcus agalactiae*, gastrointestinal tissues, immune protection

## Abstract

Neuropeptides and their corresponding receptors have been observed to play a significant role in modulating immune cell functions and epithelial barrier functions. In teleost, Neuropeptide Y (NPY) is identified to be involved in the immune regulation of intestinal tissues during bacterial infections. However, the mechanism of NPY on infected gastrointestinal tissue remains unclear, as does whether other members of the NPY family, like ligand PYYb and some receptors Y7 and Y8b, also contribute to this immunoregulatory process. Herein, the responses of PYYb and receptors Y7 and Y8b were explored after pathogen challenging, as well as the effect of NPY on the gastrointestinal tissues of Nile tilapia (*Oreochromis niloticus*). Using a NPY and *S. agalactiae* co-injection model, we measured the expression of *pyyb*, *y7*, and *y8b* and the inflammatory and oxidative stress responses in gastrointestinal tissues. *S. agalactiae* infection significantly upregulated *pyyb* expression, while co-treatment with NPY further amplified its expression. Infection caused an imbalance between *y7* and *y8b* and was alleviated by NPY. Moreover, NPY contributed to immune protection. NPY co-treatment reduced inflammatory cytokine expression in the gastrointestinal tissues and alleviated tissue damage inflicted by *S. agalactiae*. Additionally, NPY mitigated immune and oxidative stress by enhancing lysozyme, AKP and ACP activities, normalizing glucose and albumin levels, and reducing lipid peroxidation (MDA). These findings demonstrated that PYYb and receptors Y7 and Y8b were involved in the immune response caused by *S. agalactiae*. NPY not only enhanced their reactions but also reduced inflammation and oxidative stress induced by the pathogen, indicating its potential as a therapeutic intervention for *S. agalactiae* infections in fish.

## Simple Summary

Bacterial infections are a serious threat to farmed fish, causing illness, reduced growth, and even death, which can lead to significant economic losses in aquaculture. One such pathogen, *Streptococcus agalactiae*, often damages the gut of tilapia. Neuropeptide Y NPY is a highly conserved neuropeptide that plays essential roles in regulating feeding behavior, stress responses, and immune functions across vertebrates. Previous studies have demonstrated its therapeutic potential in modulating inflammatory processes and enhancing disease resistance in aquatic animals. In this study, we examined how Neuropeptide Y and related molecules, including a similar peptide called PYYb and two specific receptors, respond to infection and whether they help the fish fight disease. We found that infection increased the production of PYYb, and treatment with Neuropeptide Y further boosted this effect. Neuropeptide Y also balanced the activity of the two receptors, reduced harmful inflammation, lowered oxidative stress, and lessened tissue damage in the gut. These improvements were linked to better immune activity, healthier blood chemistry, and reduced cell damage. Our findings suggest that Neuropeptide Y could be developed as a treatment to improve disease resistance and gut health in farmed fish, offering a promising approach to controlling infections and improving the sustainability of aquaculture.

## 1. Introduction

The Neuropeptide Y (NPY) family is a group of regulatory peptides that play essential roles in various physiological processes, including appetite regulation, stress response, energy metabolism, and immune function. The family comprises three primary members: NPY, Peptide YY (PYY), and Pancreatic Polypeptide (PP) [1]. Numerous studies on the evolutionary process of the NPY family peptides have found that, early in vertebrate evolution, a single ancestral *NPY* gene gave rise to *NPY* and *PYY* through chromosomal duplication. In teleost [2], following the third round of genome duplication (3R), *NPY* and *PYY* were duplicated, resulting in *NPYa*, *NPYb*, *PYYa*, and *PYYb*. Moreover, differential losses have occurred in teleost fishes such that zebrafish (*Danio rerio*) seems to lack *NPYb* [3] and medaka (*Oryzias latipes*) lacks *PYYb* [2]. In tetrapod, the *PYY* gene underwent a local duplication event, leading to the formation of *PP* [4]. In addition, fasting has been shown to upregulate *NPYa* expression in the hypothalamus [5], whereas refeeding leads to its downregulation [6]. In contrast, *PYYb* in teleosts is predominantly expressed in the gastrointestinal tract, particularly in the foregut [7,8], while *PYYa* exhibits a primarily brain-specific expression pattern [9]. In our previous work, we cloned the members of the NPY family in Nile tilapia (*Oreochromis niloticus*) and analyzed their tissue-specific expression patterns. In the gastrointestinal tissues of tilapia, *pyyb* and its receptors *y7* and *y8b* exhibited high expression levels, suggesting that they may play a role in gastrointestinal immune responses [10].

The gastrointestinal tract of teleost serves as a critical barrier against pathogens, and its immune system plays a vital role in protecting against microbial infections [11]. Evidence from studies on mucosal vaccination and mucosal infection indicates that adaptive immune responses occur at various mucosal surfaces in teleost [12,13]. The gut of teleost serves as an important gut-associated lymphoid tissue (GALT) [14], while the stomach is also considered a potential mucosa-associated lymphoid tissue (MALT) [15]. In the gut of teleost fish, immunoglobulins (Igs) play a crucial role in defending against the invasion of potential pathogens through the epithelial barrier, particularly IgT and IgM [12]. In the gut of rainbow trout (*Oncorhynchus mykiss*) infected with *Ceratomyxa shasta*, a large number of IgT^+^ B cells have been detected, while an IgM-mediated immune response has been observed in the serum [16]. In another study on gilthead sea bream (*Sparus aurata*), increased expression of IgM was observed in the posterior intestine following infection with the *Enteromyxum leei* [17]. In addition, the number of IgM^+^ B cells in the gut of infected fish were also found to be elevated. Moreover, in rainbow trout infected with infectious pancreatic necrosis virus (IPNV), the expression levels of *IgM* and *IgT* were upregulated in the pyloric caeca [18]. Understanding the complex interactions between immune cells, signaling pathways, and microbial communities in the gut is crucial for developing strategies to enhance fish health and disease resistance.

A feedback loop between intestinal immunity and the enteric nervous system (ENS) is established through the regulation of *NPY* expression by immune cell-derived cytokines via their specific Y receptors [19]. Elevated levels of *NPY* have been reported in the serum of patients with inflammatory bowel disease (IBD) [20]. Consistently, a significant upregulation of *NPY* expression has also been observed in a dextran sulfate sodium (DSS)-induced rat model of IBD [21]. In IBD models, NPY predominantly exerts pro-inflammatory effects, with the Y1 receptor (*Y1R*) identified as a critical mediator of this response [22]. Enhanced resistance to DSS-induced colitis has been observed in both NPY-deficient mice and those treated with *Y1R* antagonists. This anti-inflammatory phenotype is characterized by functional impairments in antigen-presenting cells [23], diminished macrophage activation [24], reduced expression of pro-inflammatory cytokines such as tumor necrosis factor-alpha (*TNF-α*) and interleukin-1 (*IL-1*), and a decreased abundance of effector T cells [25]. The interaction between *NPY* and *TNF-α* in intestinal tissues appears to be tightly coordinated. In NPY knockout mice, intestinal *TNF-α* expression is significantly reduced [26]. Conversely, suppression of *NPY* expression through the administration of antisense oligodeoxynucleotides (ODNs) in rats with DSS-induced colitis not only limits leukocyte infiltration but also diminishes *TNF-α* production, thereby mitigating intestinal inflammation [25]. Additionally, *NPY* has been identified as an inducible gene within enteric neurons in both murine IBD and *Salmonella typhimurium* colitis models. *NPY* has been identified as an inducible gene in enteric neurons that promotes the expression of neuronal nitric oxide synthase (*nNOS*), subsequently triggering oxidative stress, intestinal inflammation, and gut motility disorders [27]. The full-length isoform of peptide YY (PYY_1–36_) has recently been identified as a Paneth cell-derived molecule that functions as an antifungal antimicrobial peptide, thereby contributing to the maintenance of intestinal fungal homeostasis [28]. Studies on the roles of *NPY* and *PYY* in intestinal immunity of teleost remain limited, and their specific immunological functions have yet to be elucidated.

These findings imply that neuropeptides such as *NPY* and *PYY* may become alternative strategies for improving fish immune responses and managing bacterial infections, providing a sustainable approach compared to the use of antibiotics. In this study, we treated tilapia infected with *Streptococcus agalactiae* with NPY to investigate the immune characteristics of the NPY family in gastrointestinal tissues. By understanding the underlying mechanisms by which these neuropeptides regulate immune functions in fish, more effective strategies for disease prevention and management in aquaculture might be developed, ultimately enhancing fish health and production in a more environmentally sustainable manner.

## 2. Materials and Methods

### 2.1. Experimental Fish

Nile tilapia were obtained from Guangdong Tilapia Breeding Company (Guangzhou, China). Fish were acclimatized for two weeks in a recirculating aquaculture system with a 12:12 h light/dark cycle. During the acclimation and experimental periods, water quality was continuously monitored and maintained at stable levels (water temperature: 28–29 °C; salinity: 0.02–0.04 ppt; dissolved oxygen: 6.5–7.2 mg/L; pH: 7.5–7.8; ammonia nitrogen: 0.02–0.05 mg/L; nitrite: 0.02–0.04 mg/L).During this period, they were fed a commercial diet (Qingfeng Feed Company, Foshan, China). We fed the experimental fish twice a day at 9:00 a.m. and 5:00 p.m., with each feeding amount set at 2% of the fish body weight. Subsequently, tilapia with a body weight of 25–35 g was randomly assigned to 180 L tanks, with 40 fish per tank.

### 2.2. NPY for Injection

Synthetic NPY of Nile tilapia (purity ≥95%) was obtained from ChinaPeptides Co., Ltd. (Shanghai, China), and its purity was confirmed using HPLC analysis. The amino acid sequence of tilapia NPY (Ensembl ID: ENSONIT00000005670) spans from the N-terminus to the C-terminus as follows: YPVKPENPGEDAPAEELAKYYSALRHYINLITRQRY-NH_2_ [10]. The peptide was supplied in a lyophilized form and subsequently dissolved in phosphate-buffered saline (PBS) to yield a working concentration of 100 ng/μL for intraperitoneal administration [10,29].

### 2.3. S. agalactiae Challenge

The *S. agalactiae* strain in our experiment was kindly provided by Prof. AX L, School of Life Sciences, Sun Yat-sen University [30]. The bacteria were cultured in Brain Heart Infusion (BHI, Lot. No. LM1136B, LABLEAD Inc., Beijing, China) broth in a shaking incubator at 29 °C overnight. After overnight culture, the bacterial suspension was centrifuged, and the collected cells were resuspended in PBS to a final concentration of 1 × 10^8^ CFU/mL. For infection, Nile tilapia received intraperitoneal injections of 100 μL of the prepared *S. agalactiae* suspension [31,32].

### 2.4. NPY and S. agalactiae Co-Injection Model

A total of 160 Nile tilapia were randomly divided into four groups, with 40 fish in each group. The control group was injected with 100 μL of PBS. The NPY group was injected with 100 μL of NPY working solution (100 ng/μL). The *S. agalactiae* group was injected with 100 μL of *S. agalactiae* suspension (1 × 10^8^ CFU/mL). The NPY + *S. agalactiae* group was injected with 100 μL of a premixed solution (100 ng/μL NPY + 1 × 10^8^ CFU/mL *S. agalactiae*). At 3 h, 6 h, 12 h and 24 h post-injection, 10 fish from each group were randomly selected for sampling.

All experimental procedures involving animals were conducted in strict accordance with the guidelines set by the Sun Yat-Sen University Animal Care and Use Committee, which granted approval for all protocols. (Approval No.: SYSU-IACUC-2023-B0453/2023-03/3).

### 2.5. Sample Collection

Tilapia were anesthetized with eugenol (45 mg per liter of water) [32], and serum was collected prior to decapitation after injection 24 h. The stomach, foregut, midgut, and hindgut tissues were harvested at 3 h, 6 h, 12 h and 24 h post-injection and immediately frozen in liquid nitrogen for mRNA analysis and fixed in 4% formaldehyde and subsequently embedded in paraffin for histological examination.

### 2.6. RNA Extraction and Real-Time PCR

Total RNA was isolated from frozen tissues that had been homogenized in TRIzol (Omega, Norcross, GA, USA) according to the supplier’s instructions. Following DNase I digestion (GeneStar, Beijing, China) to eliminate residual genomic DNA, first-strand cDNA was generated with the Evo M-MLV Kit (AG, Changsha, China). Subsequent qRT-PCR reactions were assembled in 10 µL volumes comprising 5 µL of 2× SYBR Green qPCR Master Mix (AG, Changsha, China). All samples were run in duplicate, and expression levels were normalized to elongation factor-1 alpha (*ef-1α*). The sequences of the oligonucleotide primers used in this study are provided in Table 1. The PCR program was 95 °C for 60 s, followed by 40 cycles of 95 °C for 15 s, 60 °C for 15 s and 72 °C for 15 s. Relative mRNA expression of the target genes was calculated using the 2^−∆∆CT^ method [32].

### 2.7. Histopathology

The stomach, foregut, midgut, and hindgut tissues of tilapia were initially fixed in 4% formaldehyde and then transferred to 70% ethanol for further processing. Tissue processing included dehydration (The dehydration procedure was as follows: 70% ethanol for 2 h, 80% ethanol for 2 h, 95% ethanol for 0.5 h, 95% ethanol for 0.5 h, 100% ethanol for 0.5 h, 100% ethanol for 1 h, 50% ethanol + 50% toluene for 1 h, toluene for 1 h, toluene for 2 h, 50% toluene + 50% paraffin for 1 h, paraffin for 2 h, and paraffin for 3 h), paraffin embedding, and sectioning to a thickness of 4 µm. The paraffin sections were stained with hematoxylin and eosin (H&E) (Beyotime, Shanghai, China) and analyzed under a Nikon light microscope at ×10 and ×20 magnification (Nikon, Tokyo, Japan).

### 2.8. Serum Biochemical Parameter Assay

Glucose (Lot. No. BC2500, Beijing Solarbio Science & Technology Co., Ltd., Beijing, China), malondialdehyde (MDA, Lot. No. AKFA013C, Beijing Boxbio Science & Technology Co., Ltd., Beijing, China) and albumin (Lot. No. AKPR039C, Beijing Boxbio Science & Technology Co., Ltd., China) in tilapia serum, as well as the enzymatic activities of alkaline phosphatase (AKP, Lot. No. A059-2-2, Nanjing Jiancheng, Nanjing, China), acid phosphatase (ACP, Lot. No. A060-2-2, Nanjing Jiancheng, China), super oxide dismutase (SOD, Lot. No. A001-1-1, Nanjing Jiancheng, China) and glutathione peroxidase (GSH-PX, Lot. No. A005-1-2, Nanjing Jiancheng, China), were determined using the corresponding commercial kits according to the manufacturer’s protocol. The activity of lysozyme was detected with a Lysozyme Assay Kit (Lot. No. E22013, Thermo Fisher, Waltham, MA, USA) following the manufacturer’s protocol.

### 2.9. Statistical Analysis

Quantitative data are presented as the mean ± SEM. Statistical analyses were conducted using SPSS 26.0 (SPSS Inc., Chicago, IL, USA). For comparisons between two groups, Student’s t-test was applied. For comparisons among multiple groups with a normal distribution, one-way ANOVA was used, while nonparametric tests were employed for other comparisons. The results were considered statistically significant at *p* < 0.05.

## 3. Results

### 3.1. S. agalactiae Infection Triggered the Expression of NPY Family in the Gastrointestinal Tissues of Tilapia

**Figure 1:**
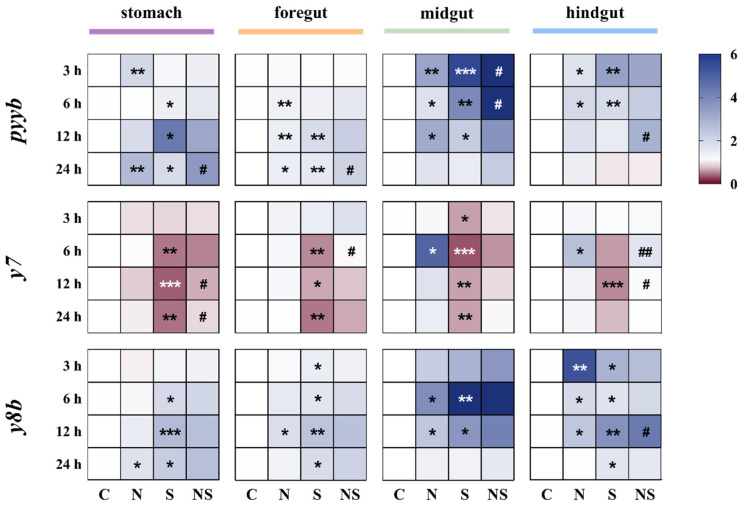
Impact of *S. agalactiae* and NPY i.p. injection on the *pyyb*, *y7* and *y8b* expression in the gastrointestinal of tilapia. C: PBS, N: NPY, S: *S. agalactiae*, NS: NPY + *S. agalactiae*. The mRNA expression levels of *pyyb*, *y7* and *y8b* in the stomach, foregut, midgut and hindgut were detected at 3 h, 6 h, 12 h and 24 h after injection. Red indicates up-regulated DEGs, and blue indicates down-regulated DEGs. Significant compared to the control group at * *p* < 0.05, ** *p* < 0.01, *** *p* < 0.001; Significant compared to the *S. agalactiae* group at # *p* < 0.05, ## *p* < 0.01, n = 10.

In the NPY and *S. agalactiae* co-injection model, the expression levels of NPY family in gastrointestinal tissues were detected (Figure 1). NPY treatment alone significantly upregulated *pyyb* expression in all four gastrointestinal segments. *S. agalactiae* infection further enhanced *pyyb* levels, with the most pronounced induction in the midgut. Notably, co-treatment with NPY markedly suppressed the infection-induced upregulation of *pyyb*, particularly in the midgut at 3 h and 6 h.

NPY treatment alone significantly increased the expression of *y7* and *y8b* in the midgut and hindgut, *y7* significantly increased at 6 h, and *y8b* significantly increased at both 6 h and 12 h. The *y7* expression substantially decreased in response to *S. agalactiae*, especially in the stomach, midgut, and hindgut from 6 h to 24 h after infection. This activation was notably reversed by NPY co-treatment, suggesting a regulatory effect of NPY on infection-induced *y7* expression. In contrast to *y7*, *S. agalactiae* challenge led to significant upregulation of *y8b*, mainly in the midgut and hindgut, with peak expression at 6–12 h. Co-treatment with NPY further enhanced *y8b* expression, particularly in the hindgut at 12 h (Figure 1).

### 3.2. Effects of NPY on Gastrointestinal Inflammatory Response in Tilapia Infected with S. agalactiae

**Figure 2:**
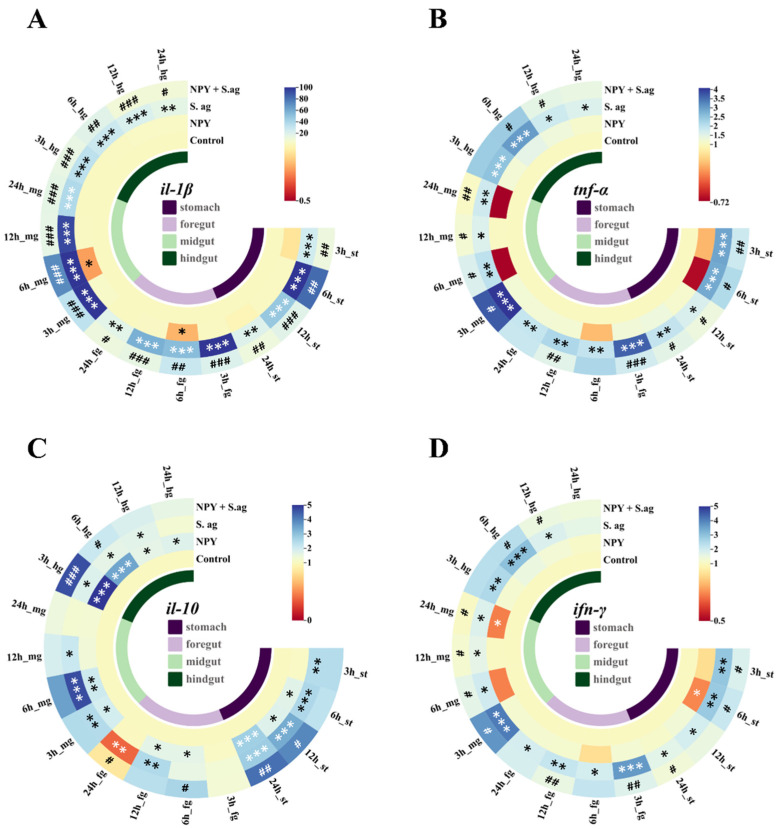
Effects of NPY on immune genes in the gastrointestinal tissues of tilapia after *S. agalactiae* infection. The mRNA expression levels of *il-1β* (**A**), *il-10* (**B**), *tnf-α* (**C**) and *ifn-γ* (**D**) in the stomach (st), foregut (fg), midgut (mg) and hindgut (hg) were detected at 3 h, 6 h, 12 h and 24 h after injection. Significant compared to the control group at * *p* < 0.05, ** *p* < 0.01, *** *p* < 0.001; Significant compared to the *S. agalactiae* group at # *p* < 0.05, ## *p* < 0.01, ### *p* < 0.001, n = 10.

We examined the expression levels of inflammatory cytokines in gastrointestinal tissues (Figure 2). NPY treatment alone modestly suppressed basal *il-1β*, *tnf-α*, and *ifn-γ* expression in the stomach, foregut, and midgut, but had no notable effect in the hindgut. Interestingly, NPY significantly downregulated *il-10* expression in all segments, particularly in the hindgut. *S. agalactiae* infection significantly upregulated *il-1β*, *tnf-α*, and *ifn-γ* mRNA levels across all intestinal segments. In contrast, il-10 was significantly elevated only in the stomach and foregut (* *p* < 0.05).

In the co-treatment group, NPY markedly attenuated the *S. agalactiae*-induced upregulation of *il-1β*, *tnf-α*, and *ifn-γ*, especially in the stomach and midgut. Concurrently, it further enhanced *il-10* expression in the stomach and hindgut (^#^ *p* < 0.05).

### 3.3. Correlation Analysis of PYY, Receptors and Inflammatory Factors in the Gastrointestinal Tract

**Figure 3:**
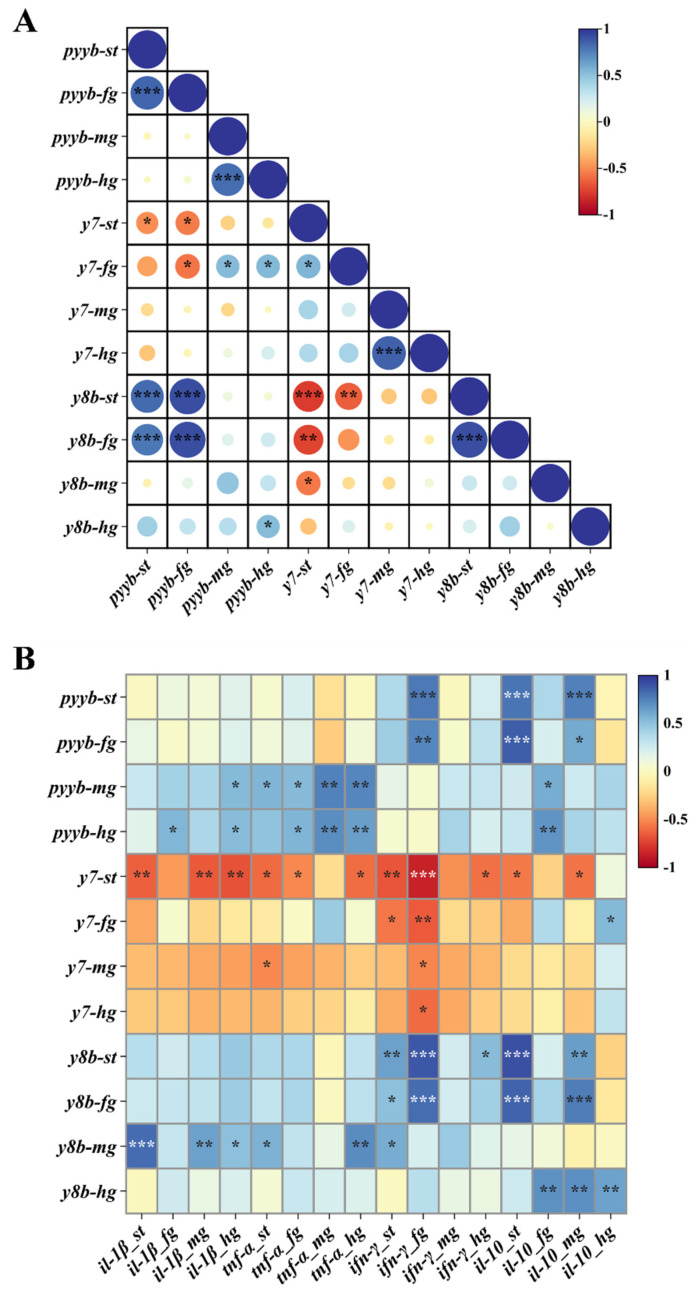
Correlation analysis between NPY family and immune genes in the stomach (st), foregut (fg), midgut (mg) and hindgut (hg). (**A**): Correlation analysis between *pyyb*, *y7* and *y8b.* (**B**): Correlation analysis between NPY family (*pyyb*, *y7* and *y8b*) and immune genes (*il-1β*, *il-10*, *tnf-α* and *ifn-γ*). Significant at * *p* < 0.05, ** *p* < 0.01, *** *p* < 0.001.

Correlation analysis of *pyyb*, *y7* and *y8b* across the four gastrointestinal tissues at different infection time points revealed that *pyyb* was significantly and negatively correlated with *y7* in the stomach and foregut, whereas it displayed a strong positive correlation with *y8b* in the same segments. In addition, *y7* and *y8b* were markedly negatively correlated (Figure 3A).

We subsequently performed correlation analyses between the temporal expression profiles of NPY family members (*pyyb*, *y8b*, *y7*) and immune genes (*il-1β*, *il-10*, *tnf-α* and *ifn-γ*) (Figure 3B). The results revealed that *pyyb* and *y8b* exhibited significant positive correlations with the changes in inflammatory cytokines across all segments. In contrast, *y7* displayed a pronounced negative correlation with the four immune genes, with the strongest inverse relationship observed in the stomach. The finding indicated that bacterial infection enhanced the expression of *pyyb* and *y8b* while concurrently suppressing *y7* in the gastrointestinal tissues of tilapia, thereby orchestrating the intestinal immune response.

### 3.4. NPY Ameliorated Gastrointestinal Tissue Damage in Tilapia Caused by S. agalactiae

**Figure 4:**
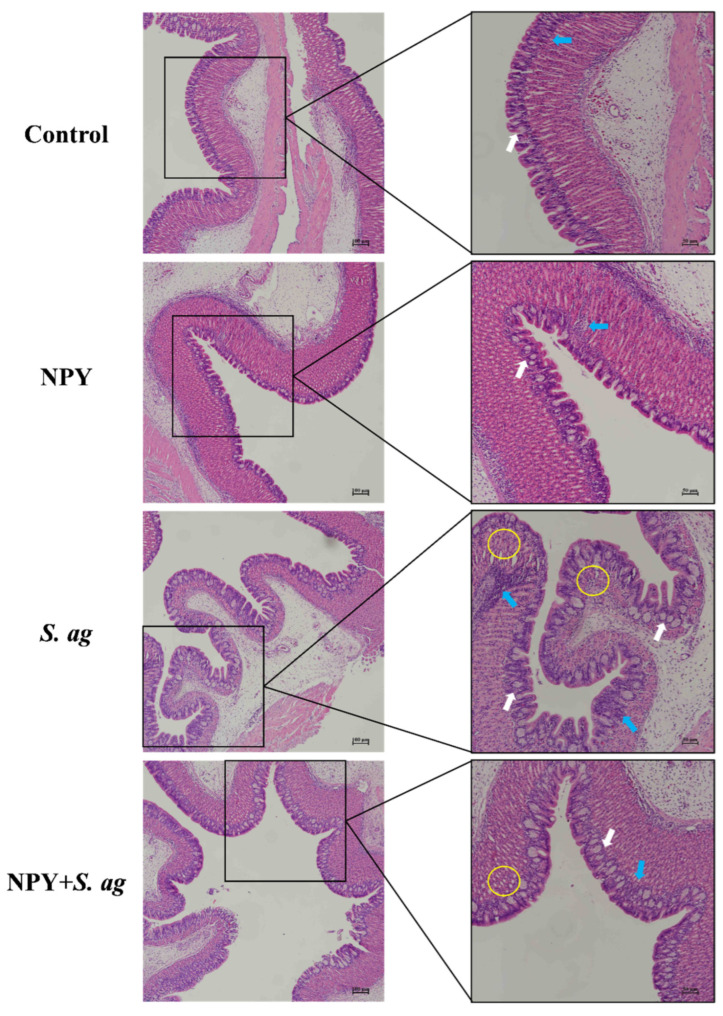
Effects of NPY injection on stomach morphology of *S. agalactiae*-infected tilapia. White arrow: surface mucus cells; yellow circle: vacuolization; blue arrow: lymphocytes. Scale bar: 100 μm (**left**) and 50 μm (**right**).

Histological analysis of gastric tissue showed that NPY treatment alone increased the number of surface mucus cells and epithelial lymphocytes. Following *S. agalactiae* infection, a marked increase in disorganized mucus cells, extensive lymphocyte infiltration, and partial epithelial necrosis was observed. In contrast, co-treatment with NPY mitigated gastric mucosal damage by reducing mucus cell numbers, improving cellular organization, and decreasing lymphocyte infiltration. Epithelial integrity was better preserved in the co-treated group (Figure 4).

**Figure 5:**
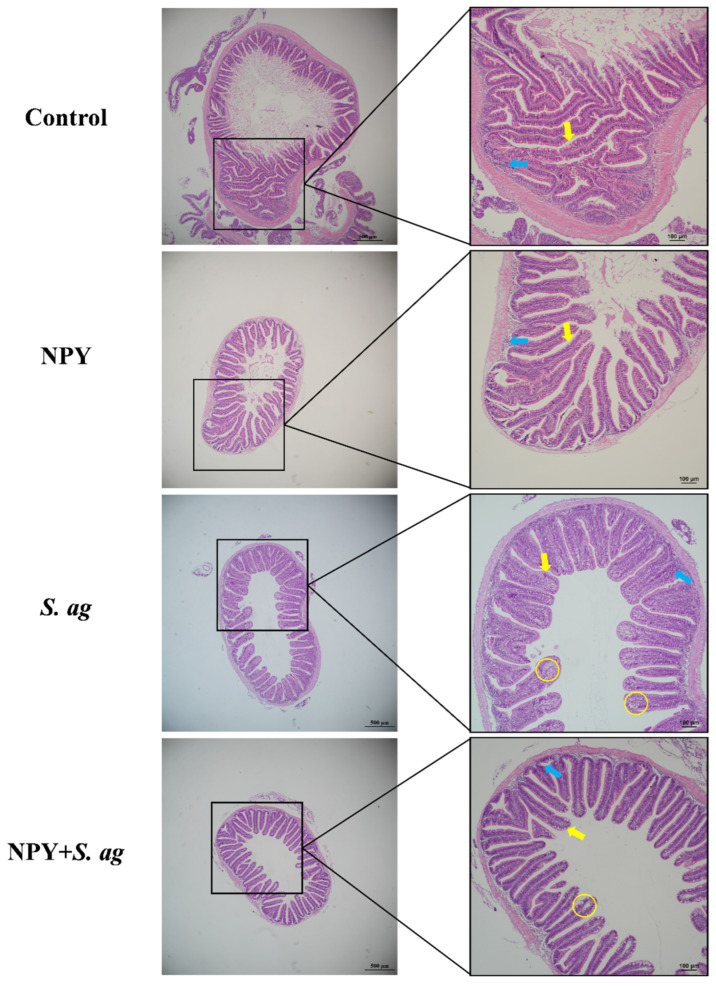
Effects of NPY injection on foregut morphology of *S. agalactiae*-infected tilapia. Yellow arrow: goblet cells; yellow circle: vacuolization; blue arrow: lymphocytes. Scale bar: 100 μm (**left**) and 50 μm (**right**).

In the foregut, NPY treatment resulted in shorter villi with an increased number of goblet cells at the villus tips, while lymphocyte levels remained unchanged. *S. agalactiae* infection led to a relatively intact intestinal wall but induced marked goblet cell hyperplasia with disorganized arrangement, severe epithelial vacuolation, and elevated intraepithelial lymphocytes. Co-treatment with NPY reduced epithelial vacuolation and lymphocyte infiltration, while goblet cell numbers remained unchanged (Figure 5).

**Figure 6:**
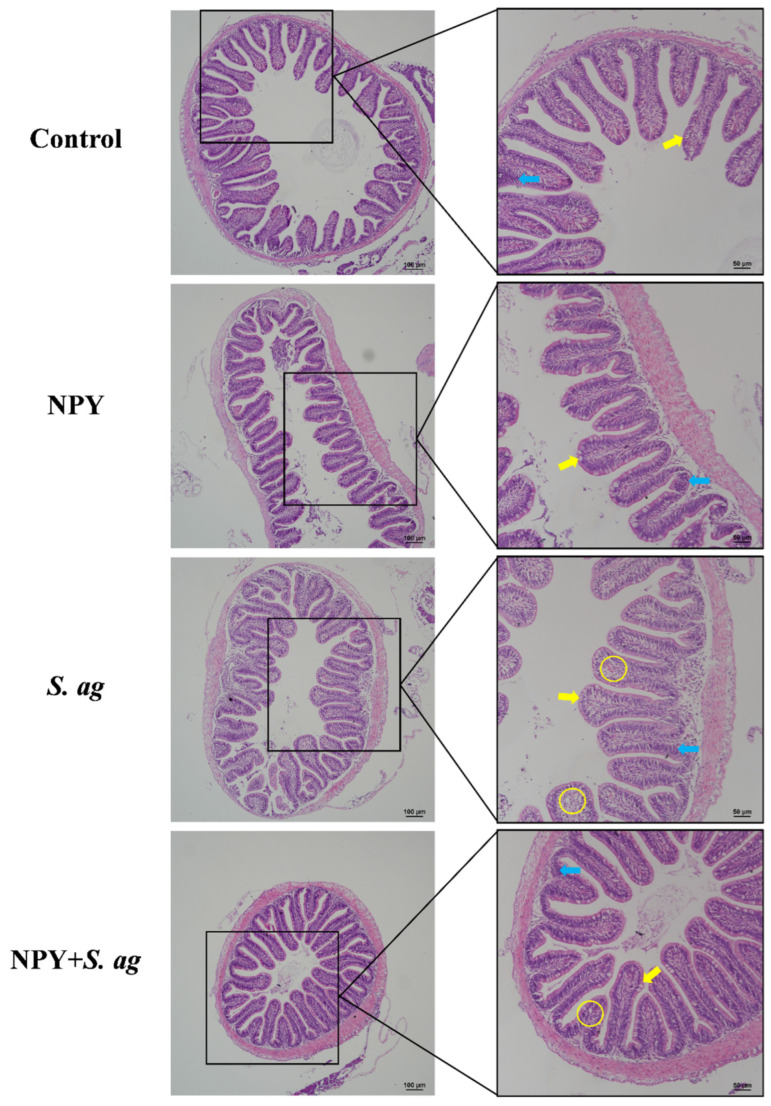
Effects of NPY injection on midgut morphology of *S. agalactiae*-infected tilapia. Yellow arrow: goblet cells; yellow circle: vacuolization; blue arrow: lymphocytes. Scale bar: 100 μm (**left**) and 50 μm (**right**).

In the midgut, NPY alone did not cause morphological alterations. In contrast, *S. agalactiae* infection disrupted the intestinal wall, causing longitudinal muscle vacuolation, disorganized goblet cell hyperplasia, epithelial vacuolation, and increased intraepithelial lymphocytes. NPY co-treatment alleviated these changes, reducing goblet cell and lymphocyte numbers as well as epithelial vacuolation (Figure 6).

**Figure 7:**
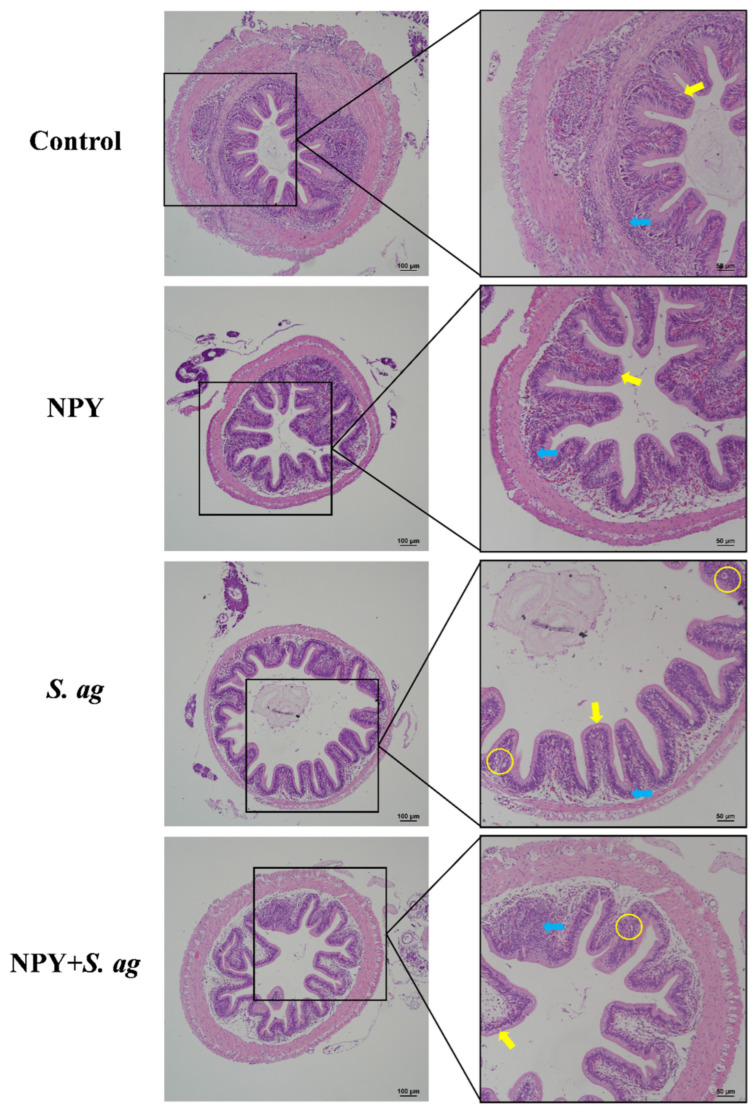
Effects of NPY injection on hindgut morphology of *S. agalactiae*-infected tilapia. Yellow arrow: goblet cells; yellow circle: vacuolization; blue arrow: lymphocytes. Scale bar: 100 μm (**left**) and 50 μm (**right**).

Similar morphological changes were observed in the hindgut. Co-treatment with NPY and *S. agalactiae* reduced vacuolation of both longitudinal muscles and epithelial cells, decreased goblet cell numbers, and had no significant effect on lymphocyte levels (Figure 7).

### 3.5. NPY Affected Immune and Oxidative Reaction Induced by S. agalactiae

**Figure 8:**
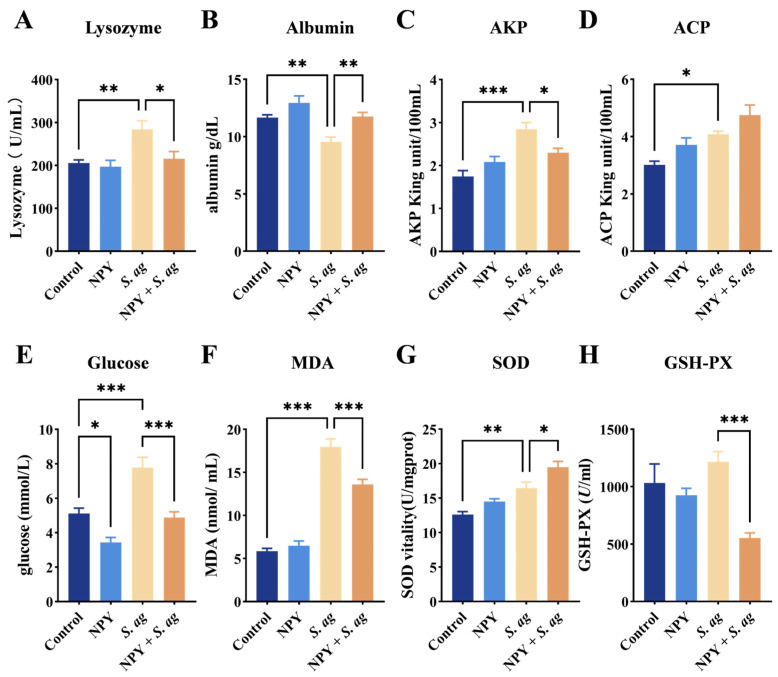
Effects of NPY on the immune and oxidative stress levels in tilapia serum caused by *S. agalactiae*. (**A**), lysozyme activity; (**B**), albumin levels; (**C**), AKP activity; (**D**), ACP activity; (**E**), glucose levels; (**F**), MDA levels; (**G**), SOD activity; (**H**), GSH-PX activity in the serum. Significant at * *p* < 0.05, ** *p* < 0.01, *** *p* < 0.001, n = 10.

The immune and oxidative stress indicators in serum were measured 24 h after infection. *S. agalactiae* infection significantly elevated lysozyme, AKP, ACP, glucose, MDA, SOD and GSH-PX activities, while markedly reducing albumin levels (Figure 8). Collectively, NPY treatment counteracted the *S. agalactiae*-induced deterioration of immunity and oxidative balance, enhancing lysozyme, AKP and ACP activities, normalizing albumin and glucose levels, and bolstering antioxidant defense (SOD and GSH-PX) while mitigating lipid peroxidation (MDA) (Figure 8).

## 4. Discussion

This study provided new insights into the immunomodulatory roles of NPY and its associated NPY-system components (*PYYb*, *Y7* and *Y8b*) in the gastrointestinal immune response of Nile tilapia under *S. agalactiae* infection. The results demonstrated that bacterial infection significantly altered the expression profiles of NPY family members in the gastrointestinal tract, characterized by upregulation of *pyyb* and *y8b*, and concurrent downregulation of *y7*. These infection-induced changes were further modulated by exogenous *NPY* administration, which exerted protective effects by suppressing inflammatory responses, maintaining tissue architecture, and restoring immune and oxidative homeostasis at the systemic level.

In mammals, previous studies have established that *NPY* plays dual roles in inflammation depending on receptor subtype and immunological context. Particularly, Y1 receptor signaling has been associated with enhanced macrophage and T cell activation, thereby aggravating inflammation in IBD models [21,33]. The upregulation of *pyyb* and *y8b* observed in infected tilapia resembled findings in rodent colitis models, where *NPY* expression was induced by pro-inflammatory mediators such as *TNF-α* and *IL-1β* [19,26,34]. Largemouth bass (*Micropterus salmoides*) injected with *NPY* exhibited suppressed lipopolysaccharide (LPS)-induced *il-1β* elevation and polyinosic-polycytidylic acid (Poly I:C)-induced *ifn-γ* elevation in the intestine [29]. This outcome aligns with the anti-inflammatory effects observed in Nile tilapia, suggesting that NPY’s intestinal immunosuppressive action is broad-spectrum and effective against multiple pathogen-associated stimuli. Furthermore, the significant positive correlation between *pyyb*/*y8b* and pro-inflammatory cytokines (*il-1β*, *tnf-α*, *ifn-γ*) supported the involvement of these NPY components in the amplification of local immune responses. In contrast, the negative correlation observed between *y7* expression and inflammatory gene expression suggested a potential regulatory or suppressive role for *y7* during infection, a dynamic requiring further mechanistic clarification.

NPY treatment alone modestly enhanced *pyyb* expression and influenced receptor gene expression in a tissue- and time-specific manner. Co-treatment with *S. agalactiae* led to a further increase in *pyyb* expression during both early and late infection phases, implying a possible feedback regulatory mechanism. This pattern aligned with earlier evidence that immune-derived cytokines can regulate *NPY* expression in GALT through receptor-specific signaling pathways [21,35,36]. Importantly, exogenous NPY administration significantly attenuated the *S. agalactiae*-induced upregulation of pro-inflammatory cytokines, especially in the stomach and hindgut, and enhanced the expression of the anti-inflammatory cytokine *il-10*. These effects reflected NPY’s known immunoregulatory activity in mammalian systems, where it suppresses pro-inflammatory signaling pathways (e.g., NF-κB) and promotes an anti-inflammatory environment [19,37]. The observed upregulation of *il-10* further indicated that NPY could facilitate the resolution phase of inflammation, contributing to immune homeostasis during bacterial infection. These results suggest that NPY modulates the intestinal immune response to *S. agalactiae* infection by suppressing pro-inflammatory cytokines and promoting anti-inflammatory *il-10* expression.

At the tissue level, *S. agalactiae* infection caused evident gastrointestinal damage, including epithelial vacuolation, goblet cells hyperplasia, and lymphocyte infiltration—hallmarks of enteritis [38,39]. Vacuolization is a common morphological feature that appears in mammalian cells following exposure to bacterial or viral pathogens, as well as to diverse natural or synthetic low-molecular-weight compounds. Vacuolization often accompanies cell death [40]. NPY administration ameliorated these histopathological changes by preserving epithelial integrity, reducing immune cell infiltration, and restoring villus morphology. Studies in largemouth bass (*Micropterus salmoides*) also found that NPY injection alleviated intestinal tissue damage induced by both LPS and Poly I:C [29]. These protective effects were consistent with studies showing that NPY contributes to maintaining gut barrier function under inflammatory conditions.

Systemically, *S. agalactiae* infection significantly disrupted immune and oxidative homeostasis, as reflected by increased serum levels of lysozyme, AKP, ACP, glucose, MDA, SOD, and GSH-PX, alongside reduced albumin concentrations. Elevated lysozyme, AKP, and ACP suggested activation of the innate immune system in response to infection, while the increase in SOD and GSH-Px represented a compensatory antioxidant response to oxidative stress [41,42]. High MDA levels, a marker of lipid peroxidation, indicated substantial oxidative damage [41]. Hypoalbuminemia, a clinical sign of systemic inflammation and liver dysfunction, further supported the severity of infection-induced stress [43]. In our previous study, immersion of juvenile tilapia in NPY solution elevated serum SOD activity and reduced lysozyme activity, thereby enhancing the juveniles’ resistance to *S. agalactiae infection* [32]. Similarly, after 18 or 30 days of immersion in tilapia recombinant NPY (trNPY), juvenile African catfish (*Clarias gariepinus*) exhibited increased body weight, along with elevated GSH levels and SOD activity, indicating an enhancement of their antioxidant defense system [44]. Remarkably, NPY administration restored these parameters: it normalized serum glucose and albumin levels, reduced MDA accumulation, and enhanced antioxidant enzyme activity, thereby mitigating oxidative injury. These results were in line with previous reports indicating the role of NPY in modulating oxidative stress and metabolic stability in both mammalian and fish models.

In summary, NPY played a dual regulatory role in the immune response of tilapia to S. agalactiae infection: it modulated the transcriptional dynamics of its own ligand-receptor system (*pyyb*, *y7*, *y8b*), suppressed excessive pro-inflammatory signaling, and restored systemic immune-metabolic balance. These findings provided strong evidence supporting the conserved immunoregulatory role of NPY in vertebrate mucosal immunity and suggested its potential as a therapeutic target in controlling bacterial infections in aquaculture species. However, the specific regulatory mechanisms of the NPY family in mucosal immunity of fish remain to be further investigated. Additionally, whether the immune-protective effects of NPY on the gastrointestinal tract are applicable to different pathogenic infections requires further study.

## 5. Conclusions

In conclusion, this study highlights the immunomodulatory and protective effects of NPY in tilapia infected with *S. agalactiae*. NPY enhances the expression of *pyyb* and regulates the expression of *y7* and *y8b* in gastrointestinal tissues, indicating its role in orchestrating the intestinal immune response. NPY treatment alleviates the inflammatory response, reduces oxidative stress, and mitigates gastrointestinal tissue damage induced by infection. These results suggest that NPY holds promise as a therapeutic agent for managing immune dysfunction and oxidative stress in aquaculture, particularly in fish species susceptible to bacterial infections such as *S. agalactiae*.

## Figures and Tables

**Table 1:** Primer sequence of qPCR.

Genes	Primer Sequence (5′-3′)	Accession Numbers	Tm	Product Size (bp)	Efficiency (%)
*ef-1α* F	ATCATTGATGCCCCTGGACA	NM_001279647.1	59	190	99.3
*ef-1α* R	CTCCAACGATGAGCTGCTTC	NM_001279647.1	59	190	99.3
*pyyb* F	GCCGTCAGGCACTATGTCAACC	XM_003438748.5	63	184	93.7
*pyyb* R	GTGTTGGTAGTGCGGGATTGTG	XM_003438748.5	62	184	93.7
*y8b* F	GCACAGCACCAATCACAACC	XM_025897149.1	60	235	99.6
*y8b* R	GCACGTGAGAATGTCTGAGC	XM_025897149.1	59	235	99.6
*il-1β* F	TGCACTGTCACTGACAGCCAA	XM_019365844.2	62	113	101.4
*il-1β* R	ATGTTCAGGTGCACTTTGCGG	XM_019365844.2	62	113	101.4
*il-10* F	CTGCTAGATCAGTCCGTCGAA	XM_013269189.3	59	94	95.3
*il-10* R	GCAGAACCGTGTCCAGGTAA	XM_013269189.3	60	94	95.3
*tnf-α* F	CTTCCCATAGACTCTGAGTAGCG	NM_001279533.1	60	161	98.2
*tnf-α* R	GAGGCCAACAAAATCATCATCCC	NM_001279533.1	60	161	98.2
*ifn-γ* F	CAACAACTCAGGCTCGCTAC	XM_001287402.1	59	167	102.5
*ifn-γ* R	TGCTCTGAACGATGTGGTCA	XM_001287402.1	59	167	102.5

## Data Availability

The data that support the findings of this study are available from the corresponding author upon reasonable request.

## Abbreviations

PP	Pancreatic Polypeptide
PYY	Peptide YY
NPY	Neuropeptide Y
ENS	Enteric Nervous System
GALT	Gut-Associated Lymphoid Tissue
IBD	Inflammatory Bowel Disease
Ig	Immunoglobulin
IgM	Immunoglobulin M
IgT	Immunoglobulin T
LPS	Lipopolysaccharide
MALT	Mucosa-Associated Lymphoid Tissue
MDA	Malondialdehyde
ACP	Acid Phosphatase
AKP	Alkaline Phosphatase
SOD	Superoxide Dismutase
GSH-PX	Glutathione Peroxidase
H&E	Hematoxylin and Eosin
OD	Optical Density
CFU	Colony-Forming Unit
IL-1	Interleukin-1
IL-10	Interleukin-10
TNF-α	Tumor Necrosis Factor Alpha
INF-γ	Interferon-gamma
nNOS	Neuronal nitric oxide synthase
qRT-PCR	Quantitative Real-Time Polymerase Chain Reaction
PBS	Phosphate-Buffered Saline
